# Characterisation and *In Vitro* Cytotoxicity of Triorganophosphinegold(I) 2-Mercaptobenzoate Complexes

**DOI:** 10.1155/MBD.1999.31

**Published:** 1999

**Authors:** Dick de Vos, Phil Clements, Simon M. Pyke, Douglas R. Smyth, Edward R. T. Tiekink

**Affiliations:** 1 Medical Department Pharmachemie B.V. Haarlem NL-2003 RN The Netherlands; 2 Department of Chemistry The University of Adelaide Adelaide 5005 Australia

## Abstract

The preparation and full NMR (^1^H, ^13^C and ^31^P) characterisation of three [R_3_PAu(2mba)] complexes, Where R = Et, Ph and Cy, and 2mba is the anion derived from 2-mercaptobenzoic acid is reported. An interesting solvent dependence in the ^1^H spectra is rationalised in terms of competing intra- and inter-molecular hydrogen bonding. An X-ray analysis of the [Ph_3_ PAu(2mba)] species reveals a linear P—Au—S arrangement and association in the lattice *via* the familar carboxylic acid dimer motif. The *in Vitro* cytotoxicity against seven human tumout lines is also described. The complexes display moderate to very high activity. Particularly noteworthy is their greater activity against the H226 cell line (non-small cell lung cancer) compared with that displayed by a range of cytotoxic drugs.


### CHARACTERISATION AND *IN VITRO* CYTOTOXICITY OF TRIORGANOPHOSPHINEGOLD(I) 2-MERCAPTOBENZOATE COMPLEXES

Dick de Vos[1], Phil Clements,[2] Simon M. Pyke,[2] Douglas R. Smyth[2] and Edward R.T. Tiekink[2,*]

[1] Medical Department, Pharmachemie B.V., NL-2003 RN Haarlem, The Netherlands
[2] Department of Chemistry, The University of Adelaide, Australia 5005

### Abstract

The preparation and full NMR ($^1H$,$^{13}C$ and $^{31}P$) characterisation of three $R_3PAu(2mba)$ complexes, where R = Et, Ph and Cy, and 2mba is the anion derived from 2-mercaptobenzoic acid is reported. An interesting solvent dependence in the $^1H$ spectra is rationalised in terms of competing intra- and inter-molecular hydrogen bonding. An X-ray analysis of the [$Ph_3PAu(2mba)$] species reveals a linear P—Au—S arrangement and association in the lattice *via* the familar carboxylic acid dimer motif. The *in vitro* cytotoxicity against seven human tumour lines is also described. The complexes display moderate to very high activity. Particularly noteworthy is their greater activity against the H226 cell line (non-small cell lung cancer) compared with that displayed by a range of cytotoxic drugs.

### Introduction

The use of gold complexes in the treatment of rheumatoid arthritis is well known [1-3]. Two classes of complexes are used in this context, namely, polymeric, water soluble gold thiolates such as *myocrisin* (sodium aurothiomalate) and *solganol* (aurothioglucose) as well as monomeric, lipophilic *auranofin* ([(1-thio-β-D-glucopyranose-2,3,4,6-tetraacetato-*S*)(triethylphosphine)gold(I)). Given their clinical use, a logical extension of the study of gold complexes is an exploration of their potential use as anti-tumour agents [*e.g.* 1-3]. As a continuation of a program investigating the anti-tumour activity of phosphinegold(I) thiolates [4,5], *i.e.* complexes containing the P—Au—S arrangement as found in *auranofin*, the *in vitro* cytotoxicity of three $R_3PAu(2mba)$ complexes is reported where R = Et, Ph and Cy, and 2mba is the anion derived from 2-mercaptobenzoic acid. The preparation of these complexes has been reported in the patent [6,7] and chemical literature [8], and the crystal structure determination of the R = Cy complex is also known [8].

### Experimental

### *General*

Analytical grade solvents were used without further purification. $^1H$ (and $^1H$-$^1H$ COSY), $^{13}C$ and $^{31}P$ NMR spectra were recorded on a Varian Gemini 2000 spectrometer operating at 300.145, 75.479 and 121.501 MHz, respectively. $^1H$-$^{13}C$ HSQC and HMBC 2D experiments were measured on a Varian INOVA NMR spectrometer operating at 599.952 and 150.873 MHz for $^1H$ and $^{13}C$ NMR, respectively. Electrospray mass spectra were obtained from methanol or ethanol solutions (NaOMe was added in the case of R = Ph and Cy (in –ve mode) in order to aid ionisation). Samples were injected into a Finagan LCQ mass spectrometer *via* direct infusion using a syringe pump. Nitrogen was used as the drying and nebulising gas. Elemental analyses were performed by Chemical and Micro Analytical Services Pty Ltd (Belmont, Victoria).

### *Preparations*

The $R_3PAuCl$ precursors were prepared according to the literature method [9] and 2-mercaptobenzoic acid (2mbaH) was purchased from Fluka. The three $R_3PAu(2mba)$ complexes were prepared using a similar procedure in each case. To a stirred ethanol solution (20 ml) of

* email: etiekink@chemistry.adelaide.edu.au

$R_3PAuCl$ (*ca* 500 mg) was added an aqueous ethanolic solution (20 ml) containing equimolar quantities of 2mbaH and KOH. The solution was stirred at room temperature for 1 h, the solvent evaporated off and the residue recrystallised from acetone solution in each case.
[$Et_3PAu$(2mba)]: Yellow crystals, Yield 82 %, m. pt 109 – 110°C. Found C, 33.23; H, 4.24. $C_{13}H_{20}AuO_2PS$ requires, C, 33.34; H, 4.30.
[$Ph_3PAu$(2mba)]: Yellow crystals, Yield 83 %, m. pt 157.5 – 159°C. Found C, 49.18; H, 3.27. $C_{25}H_{20}AuO_2PS$ requires, C, 49.03; H, 3.29. Crystals suitable for X-ray crystallography were grown from the slow evaporation and cooling of a hot ethanol solution of the complex.
[$Cy_3PAu$(2mba)]: Colourless crystals, Yield 81 %, m. pt 170 – 171°C. Found C, 47.68; H, 6.22. $C_{25}H_{38}AuO_2PS$ requires, C, 47.62; H, 6.07.

### *Crystallography for [$Ph_3PAu$(2mba)]*

Intensity data for a yellow crystal (0.13 x 0.18 x 0.34 mm) were measured at room temperature on a Rigaku AFC6R diffractometer fitted with MoK$\alpha$ radiation (graphite monochromator, $\lambda$ = 0.71073 Å) using the $\omega$:2$\theta$ scan technique so that $\theta_{max}$ was 25.0°. No decomposition of the crystal occurred during the data collection, the data set was corrected for Lorentz and polarization effects [10] and for absorption employing an empirical procedure [11] such that the minimum and maximum transmission factors were 0.352 and 1, respectively. A total of 4151 data (3942 unique) were collected and of these, 2792 that satisfied the $I \geq 3.0\sigma(I)$ criterion were used in the subsequent analysis.

**TABLE 1.** Fractional atomic coordinates for [$Ph_3PAu$(2mba)]

| Atom | *x* | *y* | *z* |
|---|---|---|---|
| Au | 0.09236(4) | 0.29464(4) | 0.35885(6) |
| S(2) | 0.3043(3) | 0.3111(3) | 0.4747(4) |
| P(1) | -0.1107(3) | 0.2895(3) | 0.2216(3) |
| O(1) | 0.1380(7) | 0.0677(7) | 0.488(1) |
| O(2) | 0.0701(8) | 0.0898(7) | 0.697(1) |
| C(1) | 0.271(1) | 0.204(1) | 0.738(1) |
| C(1') | 0.154(1) | 0.115(1) | 0.628(2) |
| C(2) | 0.3399(9) | 0.2889(9) | 0.677(1) |
| C(3) | 0.452(1) | 0.367(1) | 0.796(2) |
| C(4) | 0.492(1) | 0.357(1) | 0.958(2) |
| C(5) | 0.423(1) | 0.273(1) | 1.011(2) |
| C(6) | 0.313(1) | 0.200(1) | 0.902(1) |
| C(11) | -0.1964(9) | 0.1640(9) | 0.050(1) |
| C(12) | -0.128(1) | 0.103(1) | -0.017(1) |
| C(13) | -0.189(1) | 0.008(1) | -0.143(1) |
| C(14) | -0.321(1) | -0.027(1) | -0.208(1) |
| C(15) | -0.389(1) | 0.034(1) | -0.141(1) |
| C(16) | -0.325(1) | 0.1288(9) | -0.014(1) |
| C(21) | -0.1212(9) | 0.4173(9) | 0.141(1) |
| C(22) | -0.189(1) | 0.416(1) | -0.018(1) |
| C(23) | -0.191(1) | 0.519(1) | -0.070(1) |
| C(24) | -0.127(1) | 0.623(1) | 0.034(2) |
| C(25) | -0.057(1) | 0.626(1) | 0.195(2) |
| C(26) | -0.053(1) | 0.525(1) | 0.247(1) |
| C(31) | -0.2111(8) | 0.2808(9) | 0.348(1) |
| C(32) | -0.273(1) | 0.3638(9) | 0.374(1) |
| C(33) | -0.350(1) | 0.347(1) | 0.469(1) |
| C(34) | -0.365(1) | 0.251(1) | 0.532(1) |
| C(35) | -0.304(1) | 0.168(1) | 0.508(2) |
| C(36) | -0.226(1) | 0.184(1) | 0.417(2) |

*Crystal data:* $C_{25}H_{20}AuO_2PS$, $M$ = 612.4, triclinic, space group $P\bar{1}$, $a$ = 11.541(8) Å, $b$ = 12.081(8) Å, $c$ = 8.793(4) Å, $\alpha$ = 97.86(5)°, $\beta$ = 107.72(5)°, $\gamma$ = 102.51(6)°, $V$ = 1112(1) Å$^3$, $Z$ = 2, $D_{calc}$ = 1.828 g cm$^{-3}$, $F(000)$ = 592, $\mu$ = 68.19 cm$^{-1}$.

The structure was solved by direct-methods [12] and refined by a full-matrix least-squares procedure based on $F$ [10]. The non-hydrogen atoms were refined with anisotropic displacement parameters and hydrogen atoms were included in the model in their calculated positions (C-H 0.97 Å). The refinement was continued until convergence with sigma weights when $R$ = 0.044 and $Rw$ = 0.042. The maximum residual in the final difference map was 0.87 e Å$^{-3}$. Fractional atomic coordinates are listed in Table 1 and the numbering scheme employed is shown in Figure 3 which was drawn with ORTEP [13] at 50 % probability ellipsoids. Data manipulation was performed with the teXsan program [10] installed on an Iris Indigo workstation. Other crystallographic details, comprising fractional atomic coordinates for all atoms, thermal parameters, and all bond distances and angles (in CIF format) have been deposited at the Cambridge Crystallographic Data Centre with deposition number: 103334. Tables of observed and calculated structure factors are available on request (ERTT).

### *In vitro screening*

The standard growth medium RPMI 1640 and fetal calf serum (FCS) were obtained from Life technologies (Paisley, Scotland). Sulforhodamine B (SRB), DMSO, penicillin and streptomycin were obtained from Sigma (St Louis MO, USA), trichloroacetic and acetic acid from Baker BV (Deventer, NL) and PBS from NPBI BV (Emmer-Compascuum, NL).

The test and reference compounds were dissolved to a concentration of 238095 ng/ml in full medium, by 21 fold dilution of a DMSO solution which contained 1 mg compound/200 µl. DMSO was used as the solvent owing to poor solubility in ethanol. Further, the [$Cy_3PAu$(2mba)] complex was heated at 37°C for 1 h to effect solubility. Cytotoxicity was estimated by the microculture SRB test [14]. On day 0, 150 µl trypsinised tumour cells (1500 – 2000 cells/well) were plated in 96 wells flatbottom microtiter plates (falcon 3072, BD). The plates were preincubated for 48 h at 37°C, 8.5 % $CO_2$ to allow the cells to adhere. On day 2, a three fold dilution sequence of ten steps was made in full medium, starting with the 238095 ng/ml stock solution. Every dilution was used in quadruplicate by adding 50 µl to a column of four wells. This results in a highest concentration of 59523 ng/ml present in column 12. Column 2 was used for the blank. To column 1 phosphate buffered saline (PBS) was added to diminish interfering evaporation. On day 7, the incubation was terminated by washing the plate twice with PBS. Subsequently, the cells were fixed with 10 % trichloroacetic acid in PBS and placed at 4°C for 1 h. After five washings with tap water, the cells were stained for at least 15 minutes with 0.4 % SRB dissolved in 1 % acetic acid. After staining, the cells were washed with 1 % acetic acid to remove unbound stain. The plates were air dried and the bound stain was dissolved in 150 µl 10mM Tris-base. The absorbance was read at 540 nm using an automated microplate reader (Labsystems Multiskan MS). Data were used for the construction of concentration-response curves and determination of $ID_{50}$ values by use of Deltasoft 3 software.

The following human tumour cell lines were used: MCF7 (breast cancer), EVSA-T (breast cancer), WIDR (colon cancer), IGROV (ovarian cancer), M19 MEL (melanoma), A498 (renal cancer) and H226 (non-small cell lung cancer). The WIDR, IGROV, M19 MEL, A498 and H226 cell lines are found on the currently used anti-cancer screening panel of the National Cancer Institute, USA [15]. The MCF7 cell line is estrogen receptor (ER)+/progesterone receptor (PgR)+ and the cell line EVSA-T is (ER)-/(PgR)-.

Prior to the experiments a mycoplasma test was performed on all the cell lines and found to be negative. All cell lines were maintained in a continuous logarithmic culture in RPMI 1640 medium with Hepes and phenol red. The medium was supplemented with 10 % FCS, penicillin 100 IU/ml streptomycin 100 µg/ml. The cells were mildly trypsinised for passage and for use in the experiments.

**TABLE 2.** $^1$H NMR spectra (δ in ppm, coupling constants, in Hertz, are given in parentheses) recorded in $CDCl_3$ and DMSO-$d_6$ solutions for 2mbaH and $R_3PAu$(2mba). Abbreviations: d = doublet, t = triplet, dd = doublet of doublets, dt = doublet of triplets, dq = doublet of quartets, dtt = doublet of triplet of triplets, br = broad, u = unobserved. The atom lables for the thiol/thiolate is shown in the scheme. The labels α to δ refer to the nuclei of the phosphine ligands.

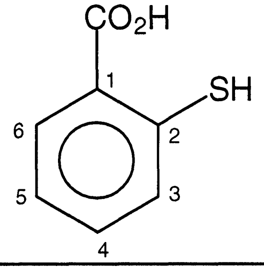

| | 2mbaH* | | R = Et | | R = Ph | | R = Cy | |
|---|---|---|---|---|---|---|---|---|
| | $CDCl_3$ | DMSO-$d_6$ | $CDCl_3$ | DMSO-$d_6$ | $CDCl_3$ | DMSO-$d_6$ | $CDCl_3$ | DMSO-$d_6$ |
| H3 | 7.31 dd (7.8, 1.2) | 7.49 dd (8.1, 0.9) | 7.50 dd (7.7, 1.4) | 7.65 dd (8.1, 1.2) | 7.74 dd (7.8, 1.2) | 7.76 d-br (7.5) | 7.65 dd (7.6, 1.8) | 7.66 d-br (7.8) |
| H4 | 7.35 ddd (7.5, 7.5, 1.4) | 7.35 ddd (7.8, 7.8, 1.7) | 7.38 ddd (7.4, 7.4, 1.6) | 7.11 ddd (7.6, 7.6, 1.6) | 7.30 ddd (7.5, 7.5, 1.8) | 7.13 dd- br (7.2) | 7.28 ddd (7.5, 7.5, 1.5) | 7.10 ddd (7.6, 7.6, 1.5) |
| H5 | 7.10 ddd (7.5, 7.5, 1.2) | 7.16 ddd (7.5, 7.5, 1.1) | 7.19 ddd (7.7, 7.7, 1.3) | 6.95 ddd (7.4, 7.4, 1.3) | 7.20 ddd (8.1, 8.1, 1.5) | 6.99 dd-br (7.4) | 7.17 ddd (7.5, 7.5, 1.5) | 6.95 ddd (7.6, 7.6, 1.5) |
| H6 | 8.12 dd (7.8, 1.8) | 7.91 dd (8.0, 1.4) | 8.30 dd (7.5, 1.2) | 7.34 dd (7.5, 1.5) | 8.32 dd (8.0, 1.7) | 7.38 dd (7.4, 1.4) | 8.32 dd (7.8, 1.6) | 7.36 dd (7.8, 1.8) |
| OH | u | 13.01 br | u | 12.58 br | 13.39 br | 12.72 br | 13.49 br | 12.50 br |
| (P) Hα | | | 1.83 dq [b] (10.1, 7.4) | 1.92 dq [b] (10.2, 7.5) | | | 1.98 dt-br [a] (10.8, 13.4) | 2.17 dtt [a] (9.5, 12.0, 3.0) |
| Hβ | | | 1.17 dt [c] (18.9, 7.7) | 1.11 dt [c] (18.7, 7.7) | 7.43-7.49 m | 7.49-7.56 m | 1.84 d-br [e] (12.6) 1.28 m [a] | 1.78 d-br [e] (12.3) 1.33 dtt [a] (12.6, 12.6) |
| Hγ | | | | | 7.43-7.49 m | 7.54-7.58 m | 1.90 d-br [e] (10.2) 1.39 m [a] (12.6) | 1.95 d-br [e] (10.8) 1.42 dtt [a] (11.4, 12.3, 3.9) |
| Hδ | | | | | 7.50-7.54 m | 7.58-7.62 m | 1.72 d-br [e] (11.4) 1.20 m [a] | 1.65 d-br [e] (12.0) 1.21 dtt [a] (12.6, 12.6, 3.2) |

[a, e] Data for the axial and equatorial $^1$H, respectively at 599.952 MHz; For $H_\alpha$: ($^2J_{H\text{-}P}$, $^3J_{Ha\text{-}Ha}$, $^3J_{Ha\text{-}He}$), $H_\beta$ (where appropriate): ($^2J_{Ha\text{-}He}$, $^3J_{Ha\text{-}Ha}$), $H_\gamma$ (where appropriate): ($^2J_{Ha\text{-}He}$, $^3J_{Ha\text{-}Ha}$, $^3J_{Ha\text{-}He}$), $H_\delta$ (where appropriate): ($^2J_{Ha\text{-}He}$, $^3J_{Ha\text{-}Ha}$, $^3J_{Ha\text{-}He}$).

[b] ($^2J_{H\text{-}P}$, $^3J_{H\text{-}H}$).

[c] ($^3J_{H\text{-}P}$, $^3J_{H\text{-}H}$).

* SH: 4.61 sharp ($CDCl_3$), 5.33 broad (DMSO-$d_6$).

### Results and Discussion

The three $R_3PAu$(2mba), R = Et, Ph and Cy, complexes display IR characteristics consistent with an earlier report [8]. Full asignment of $^1$H and $^{13}$C chemical shifts, obtained in both $CDCl_3$ and

DMSO-d6 solutions, are listed in Tables 2 and 3. The assignments were based on the interpretation of COSY, HSQC and HMBC methodologies. Interestingly, a solvent dependence was found. Notably, in the $^{1}H$ NMR spectra the positions of the chemical shifts due to H4 and H5 nuclei were variable as were the C3-C6 resonances in the $^{13}C$ NMR spectra.

Figure 1 displays major solvent dependence in the chemical shift of H6 in the spectra of [$Cy_3PAu(2mba)$], with only minor solvent dependence observed for H3-H5. In 100 % $CDCl_3$, Figure 1(a), H6 appears downfield at 8.32 ppm due to anisotropy of the adjacent carbonyl $\pi$-bond. However, in 100 % DMSO-$d_6$ , Figure 1(g), H6 is shifted upfield to 7.36 ppm, a shift of almost 1 ppm. This dramatic shift was proven by the gradual titration of DMSO-$d_6$ into a $CDCl_3$ solution of [$Cy_3PAu(2mba)$] as shown in spectra Figure 1(b) -1(f). This dramatic shift could be caused by a conformational flip of the -$CO_2H$ group induced by introduction of polar DMSO-$d_6$ into the solvation sphere of the [$Cy_3PAu(2mba)$] molecule. Figure 2 shows the proposed structures of [$Cy_3PAu(2mba)$] in 100 % $CDCl_3$ (a) and 100 % DMSO-$d_6$ (b).

In $CDCl_3$ (a), the anisotropic effects of the carbonyl $\pi$-bond are facilitated by the intramolecular O-H...S hydrogen bonding locking the -$CO_2H$ moiety into position. The 1 ppm shift clearly indicates that this orientation does not also exist in DMSO-$d_6$, i.e. H6 is no longer in the deshielding region of the carbonyl $\pi$-bond. The polar DMSO-$d_6$ molecules must disrupt the intramolecular hydrogen bonds with the formation of O-H...DMSO-$d_6$ intermolecular hydrogen bonds facilitated by bond rotation around the C-C bond joining the phenyl ring to the -$CO_2H$ moiety, as shown in Figure 2 (b). This bond rotation may also facilitate a weak Au...O contact as there is no evidence of a conformational flip for 2mba with only a 0.21 ppm solvent dependant shift of H6 observed. However, the possibility of a weak Au...O interaction is difficult to investigate as $^{197}Au$ (100% abundance) is not a suitable isotope to probe the structure of gold complexes.

**TABLE 3.** $^{13}C$ NMR spectra ($\delta$ in ppm, coupling constants, in Hertz, are given in parentheses) recorded in $CDCl_3$ and DMSO-$d_6$ solutions for 2mbaH and $R_3PAu(2mba)$. Abbreviation: d = doublet. Atom labels as for Table 2.

| | 2mbaH | | Et | | Ph | | Cy | |
|---|---|---|---|---|---|---|---|---|
| | $CDCl_3$ | DMSO-$d_6$ | $CDCl_3$ | DMSO-$d_6$ | $CDCl_3$ | DMSO-$d_6$ | $CDCl_3$ | DMSO-$d_6$ |
| C1 | 124.8 | 126.5 | 131.4 | 135.6 | 131.3 | 135.7 | 131.1 | 135.3 |
| C2 | 139.3 | 138.1 | 139.0 | 142.4 | 138.7 | 141.2 | 139.5 | 142.4 |
| C3 | 131.1 | 130.9 | 137.4 | 134.8 | 137.5 | 135.1 | 137.2 | 134.5 |
| C4 | 133.3 | 132.4 | 131.4 | 128.9 | 131.5 | 129.0 | 131.2 | 128.6 |
| C5 | 124.8 | 124.6 | 125.5 | 122.9 | 125.7 | 123.1 | 125.3 | 122.7 |
| C6 | 132.7 | 131.4 | 133.0 | 128.5 | 133.1 | 128.4 | 133.0 | 128.5 |
| C=O | 171.5 | 167.6 | 168.2 | 169.8 | 168.2 | 169.7 | 168.1 | 169.4 |
| Cα | | | 17.8 d | 17.8 d | 128.7 d (58.4) | 128.9 d (55.9) | 33.2 d (28.1) | 32.4 d (28.9) |
| Cβ | | | 8.9 | 8.9 | 134.1 d (13.7) | 133.8 d (13.6) | 26.9 d (12.1) | 26.3 d (12.0) |
| Cγ | | | | | 129.3 d (11.4) | 129.6 d (11.2) | 30.8 | 30.3 |
| Cδ | | | | | 132.0 d (2.6) | 132.0 d (2.2) | 25.7 | 25.4 |

Since the $^{1}H$ resonances of the 2mba ligand of the three $R_3PAu(2mba)$ complexes are very similar in $CDCl_3$ and also in DMSO-$d_6$, it is concluded that Et and Ph derivatives have similar solution structures to that proposed [$Cy_3PAu(2mba)$].

In $CDCl_3$ solution the $^{31}P$ NMR chemical shifts were $\delta$ 37.1, 37.6 and 58.2 ppm, respectively and in DMSO-$d_6$ solution they were $\delta$ 39.7, 38.2 and 57.5 ppm, respectively. These resonances are in the range expected for complexes of this type [16]. Electrospray mass spectroscopic analysis in the negative ion mode revealed $[M-H]^-$ in each case. For R = Et, the relative abundance was 48 % and for the remaining complexes this ion was the only ion present.

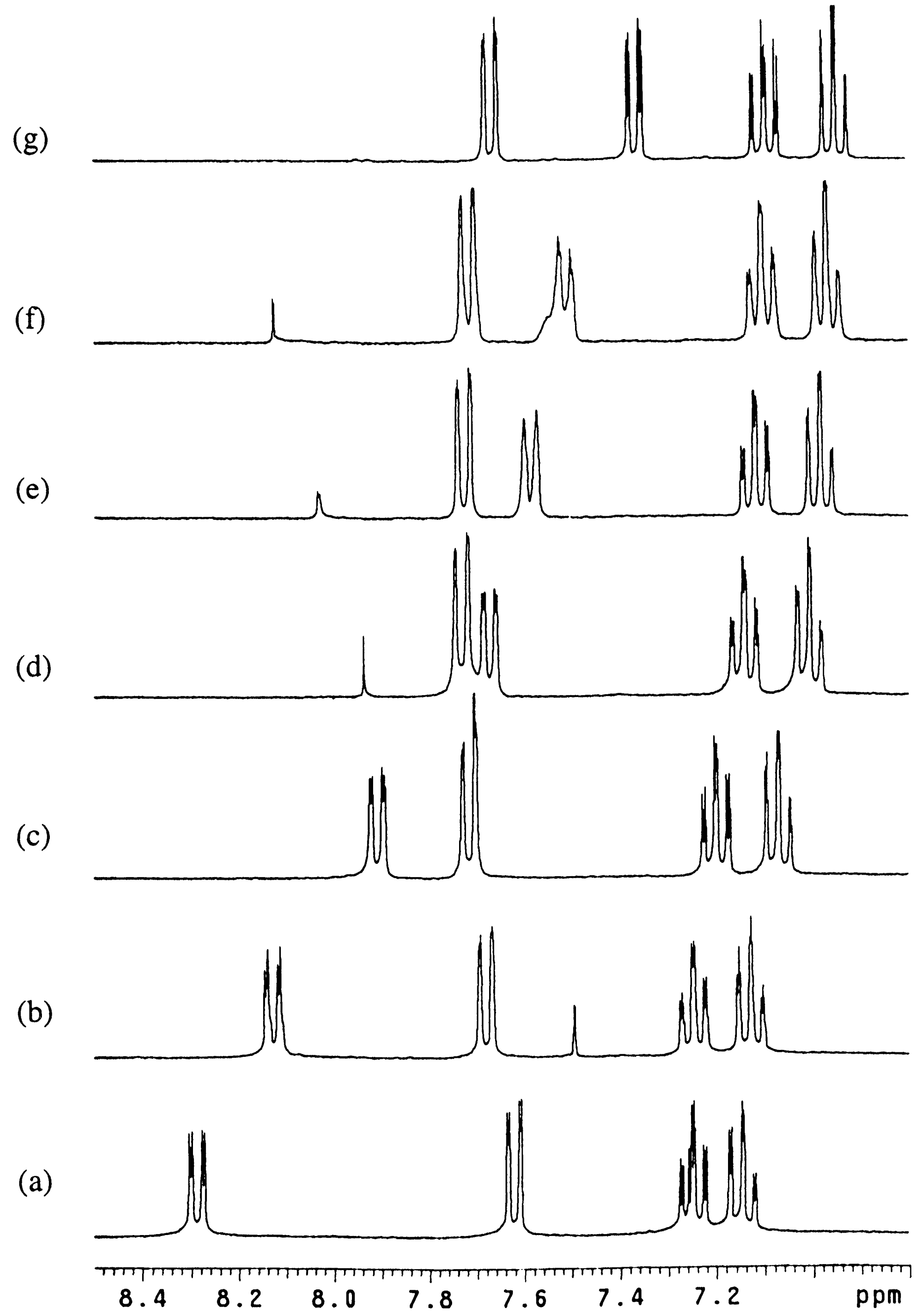

**Figure 1.** The aromatic region of 300 MHz $^1H$ spectra of $[Cy_3PAu(2mba)]$ in 100 % $CDCl_3$ (a); in $CDCl_3$/DMSO-$d_6$ mixtures of 400μl:50μl (b), 400μl:100μl (c), 400μl:200μl (d), 400μl:300μl (e), 400μl:400μl (f); and in 100 % DMSO-$d_6$

The spectrum for the R = Et complex also showed a m/e at 503 which was assigned to $[Au(2mba)_2]^-$ (100 %). For the R = Ph and Cy complexes a peak at 249 (91 % and 49 %, respectively) was assigned to $[(2mba)Au(2mba\text{-}H)]^{2-}$. In the positive mode, only ions were found in the spectrum of the R = Et complex with the major ions, *i.e.* m/e 783, assigned to $[2mba(AuPEt_3)_2]^+$ (100 %) and m/e 433, assigned to $[Au(PEt_3)_2]^+$ (24 %). Crystals for the R = Ph complex were obtained and hence, an X-ray crystal structure analysis was performed.

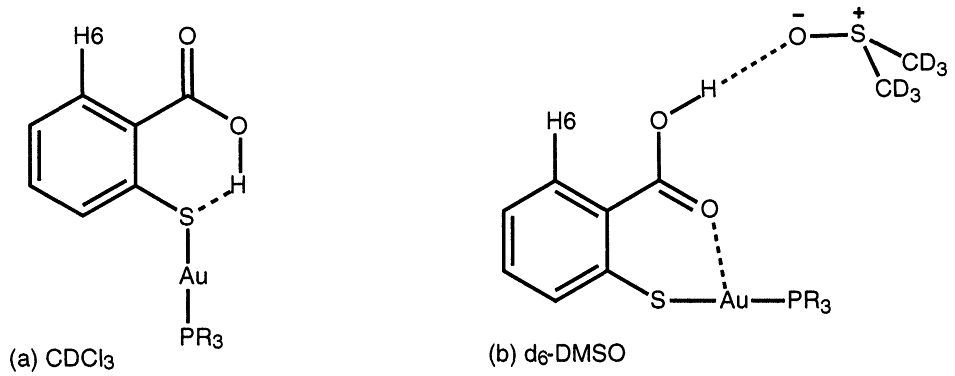

**Figure 2.** Proposed solution structures of $[Cy_3PAu(2mba)]$ in $CDCl_3$ and DMSO-$d_6$ solution.

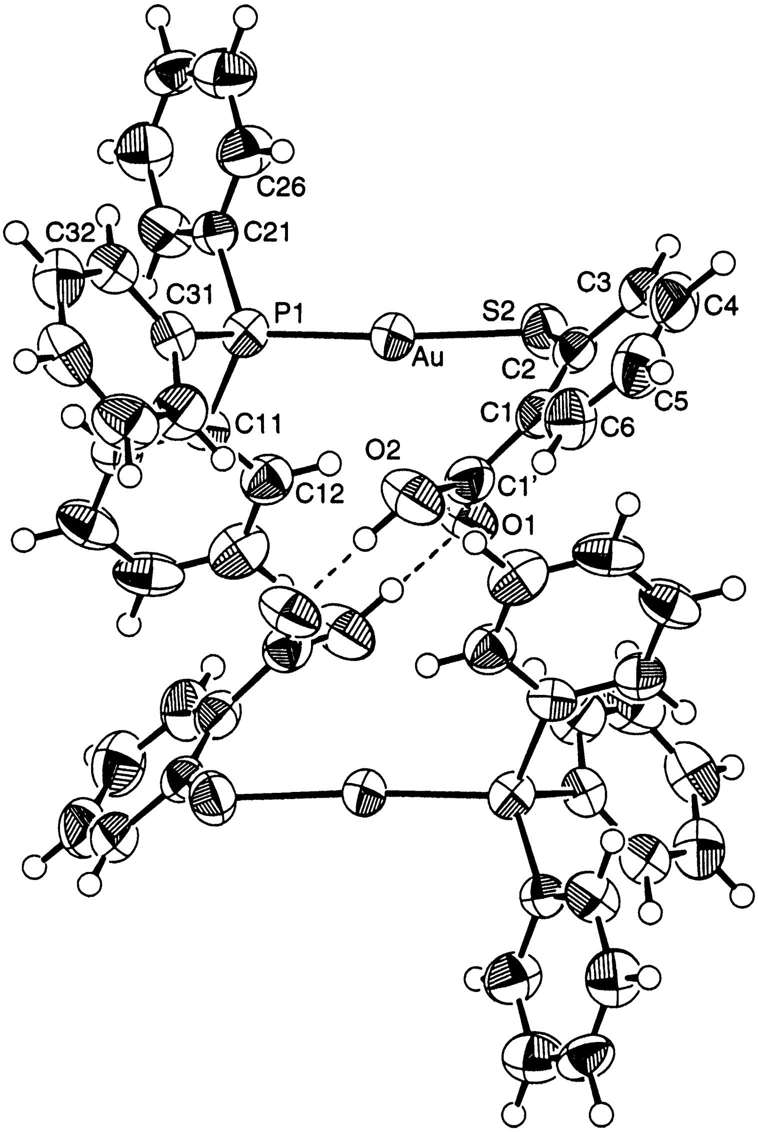

**Figure 3.** The molecular structure of $[Ph_3PAu(2mba)]$ showing the association between centrosymmetrically related molecules

### *Molecular structure of [Ph$_3$PAu(2mba)]*

The molecular structure of [Ph$_3$PAu(2mba)] is illustrated in Figure 3 and selected interatomic parameters are collected in Table 3.

The gold atom exists in an almost linear geometry defined by a sulfur atom derived from the 2mba anion and the phosphorus atom; S—Au—P is 173.1(1)°. The Au—S and Au—P distances of 2.298(3) Å and 2.269(3) Å, respectively are comparable to the equivalent distances of 2.313(1) Å and 2.271(1) Å, respectively found in the R = Cy analogue [8]. As can be noted from Figure 1 the carboxyl residue is orientated so as to place the carbonyl O(1) atom in close proximity to the gold atom. The Au...O(1) separation of 3.193(8) Å is close to the sum of the van der Waals radii of these atoms of 3.20 Å [17] and thus, does not represent a significant bonding interaction. The 2mba ligand is not planar as seen in the C(2)/C(1)/C(1')/O(1) torsion angle of 37(2)°. Further twisting about the C(1)—C(1') bond could result in a tighter interaction between the gold and O(1) atoms, however, this would result is less effective intermolecular interactions in the lattice. In the lattice, the familiar carboxylate dimer configuration is found such that H(2O)...O(1)$^i$ is 1.65 Å, O(1)...O(2)$^i$ is 2.61(1) Å and O(2)—H(2O)...O(1)$^i$ is 172°, i.e. $R_2^2(8)$ [18], (symmetry operation *i*: -*x*, -*y*, 1-*z*). There are no Au...Au contacts less than 6.0 Å in the lattice. Interestingly, in the R = Cy structure [8], an alternative conformation of the 2mba ligand is found such that the carboxyl residue is directed away from the gold centre and is best described as adopting an *exo* conformation as opposed to a *endo* conformation as for the R = Ph structure; see Figure 4.

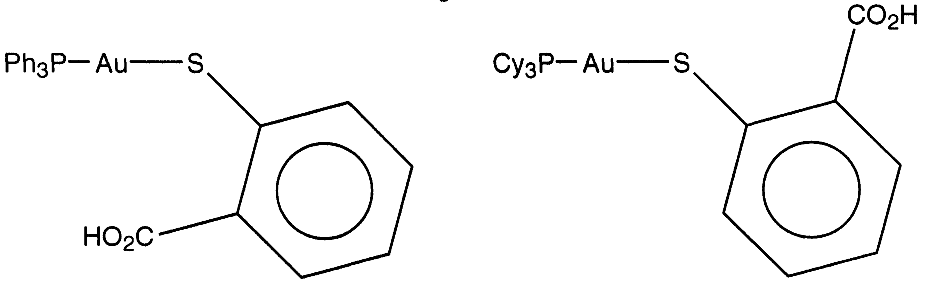

**Figure 4.** Alternate conformations found in the R$_3$PAu(2mba) molecules in the solid state as established by X-ray crystallography

**TABLE 3.** Selected interatomic parameters (Å, deg.) for [Ph$_3$PAu(2mba)]

| | | | |
|---|---|---|---|
| Au—S(2) | 2.298(3) | Au—P(1) | 2.269(3) |
| S(2)—C(2) | 1.77(1) | P(1)—C(11) | 1.83(1) |
| P(1)—C(21) | 1.80(1) | P(1)—C(31) | 1.83(1) |
| S(2)—Au—P(1) | 173.1(1) | Au—S(2)—C(2) | 110.5(3) |
| Au—P(1)—C(11) | 114.8(3) | Au—P(1)—C(21) | 112.0(3) |
| Au—P(1)—C(31) | 113.7(3) | C(11)—P(1)—C(21) | 107.0(5) |
| C(11)—P(1)—C(31) | 103.0(5) | C(21)—P(1)—C(31) | 105.5(5) |

The effect on the gold atom geometry of the intramolecular Au...O(1) interaction in the R = Ph structure is minimal with the major influence being a greater distortion from linearity, *i.e.* S—Au—P is 173.1(1)° *cf.*176.8(1)° for the R = Cy complex. Allied with the weak nature of the Au...O(1) interaction is the expansion of Au—S(2)—C(2) angle to 110.5(3) *cf.* 102.8(1); a contraction of the Au—S(2)—C(2) angle would result in a closer Au...O(1) interaction. Similar variations in structure have been reported for related R$_3$PAu(S$_2$COR') structures for which the behaviour was rationalised in terms of steric interference/optimal crystal packing rather than electronic effects associated with the phosphine ligands [19,20].

### Antitumour Testing

The cytotoxicity of the R$_3$PAu(2mba), R = Et, Ph and Cy, complexes was tested *in vitro* against seven well-characterised human tumour cell lines and the microculture sulforhodamine B (SRB) test (see Experimental). Results are listed in Table 4. The R$_3$PAu(2mba), R = Et, Ph and Cy, complexes display a very high to a moderate cytotoxicity ($ID_{50}$ < 250 ng/ml to 250 – 2500 ng/ml). Generally, amongst the gold complexes, the R = Et derivatives are the most active against the range of cell lines tested and the R = Ph complexes, the least active, however, the relatively small range of $ID_{50}$ values is noted. Notable is the observation that the gold complexes are significantly more active than cisplatin against the A498 (renal cancer) and H226 (non-small cell lung cancer) cell lines. Indeed, the gold complexes were more active than any of the doxorubicin, cisplatin, 5-fluorouracil, methotrexate and etoposide drugs against the H226 cell line. This result may indicate some selectivity in activity and it will be of some interest to determine whether the observed *in vitro* activity is maintained *in vivo*.

**TABLE 4.** *In vitro* $ID_{50}$ values (ng/ml) for the $R_3PAu(2mba)$, R = Et, Ph and Cy, complexes and new data for some established cytotoxic drugs: doxorubicin (DOX), cisplatin (CPT), 5-fluorouracil (5-FU), methotrexate (MTX) and etoposide (ETO)

| Compound | cell line | | | | | | |
|---|---|---|---|---|---|---|---|
| | MCF7 | EVSA-T | WIDR | IGROV | M19 MEL | A498 | H226 |
| R = Et | 405 | 110 | 984 | 43 | 118 | 65 | 69 |
| R = Cy | 411 | 225 | 772 | 182 | 292 | 95 | 97 |
| R = Ph | 791 | 356 | 982 | 117 | 298 | 151 | 173 |
| DOX | 10 | 8 | 11 | 60 | 16 | 90 | 199 |
| CPT | 699 | 422 | 967 | 169 | 558 | 2253 | 3269 |
| 5-FU | 750 | 475 | 225 | 297 | 442 | 143 | 340 |
| MTX | 18 | 5 | <3 | 7 | 23 | 37 | 2287 |
| ETO | 2594 | 317 | 150 | 580 | 505 | 1314 | 3934 |

The cell lines mentioned in this contribution have been used extensively in the analysis of the cytotoxicity of various organotin compounds [*e.g.* 21 - 23 and see review [24]. It is pertinent to note that, often, the *in vitro* activity of the studied organotin compounds is much greater than that displayed by the $R_3PAu(2mba)$ complexes described herein.

**Acknowledgements**
The University of Adelaide is thanked for the award of a Postgraduate Research Award to D.R.S. and the Australian Research Council is thanked for support of the crystallographic facility.

**Note Added in Proof**
During the final preparation of this paper, a related paper reporting some NMR work and the X-ray crystal structure of $[Ph_3PAu(2mba)]$ appeared [25].

**References**
1. R.V. Parish, *Interdisciplinary Sci. Rev.*, 1992, **17**, 221.
2. R.J. Sue and P.J. Sadler, *Metal-Based Drugs*, 1994, **1**, 107.
3. E.R.T. Tiekink and M.W. Whitehouse, in Handbook of Metal-Ligand Interactions in Biological fluids. G. Berthon (Ed.) Marcel Dekker, Inc., Vol. 2, 1995, 1266.
4. E.R.T. Tiekink, P.D. Cookson, B.M. Linahan and L.K. Webster, *Metal-Based Drugs*, 1994, **1**, 299.
5. L.K. Webster, S. Rainone, E. Horn and E.R.T. Tiekink, *Metal-Based Drugs*, 1996, **3**, 63.
6. B.M. Sutton and J. Weinstock, US Patent 3842107 15 Oct., 1974; *Chem. Abs.*, **82**(9), 57937h.
7. B.M. Sutton and J. Weinstock, US Patent 73-381832 23 July, 1973; *Chem. Abs.*, **84**(3), 17559w.
8. P.D. Cookson and E.R.T. Tiekink, *J. Coord. Chem.*, 1992, **26**, 313.
9. A.K. Al-Saády, C.A. McAuliffe, R.V. Parish and J.A. Sandbank, *Inorg. Syn.*, 1985, **23**, 191.

10. *teXsan, Single Crystal structure analysis software*, Version 1.6 (1993), Molecular Structure Corporation, The Woodlands, TX, USA.
11. N. Walker and D. Stuart, *Acta Crystallogr.*, 1983, *A***39**,158.
12. P.T. Beurskens, G. Admiraal, G. Beurskens, W.P. Bosman, S. García-Granda, J.M.M. Smits and C. Smykalla, The DIRDIF program system. Technical report of the crystallography laboratory, University of Nijmegen, The Netherlands, 1992.
13. C.K. Johnson, ORTEP. Report ORNL-5138 (1976), Oak Ridge National Laboratory, TN, USA.
14. Y.P. Kepers, G.J. Peters, J. van Ark-Otte, B. Winograd and H.M. Pinedo, *Eur. J. Cancer*, 1991, **27**, 897.
15. M.R. Boyd, *Principles and Practice of Oncology*, 1989, **3**, 1.
16. P.D. Cookson, E.R.T. Tiekink and M.W. Whitehouse, *Aust. J. Chem.*, 1994, **47**, 577.
17. A. Bondi, *J. Phys. Chem.*, 1964, **68**, 441.
18. M.C. Etter, J.C. MacDonald and J. Bernstein, *Acta Crystallogr.*, 1990, B**46**, 256.
19. G. Siasios and E.R.T. Tiekink, *Z. Kristallogr.*, 1993, **204**, 95.
20. G. Siasios and E.R.T. Tiekink, *Z. Kristallogr.*, 1993, **205**, 261.
21. D. de Vos, R. Willem, M. Gielen, K.E. van Wingerden and K. Nooter, *Metal-Based Drugs*, 1998, **5**, 179.
22. M. Kemmer, M. Gielen, M. Biesemans, D. de Vos and R. Willem, *Metal-Based Drugs*, 1998, **5**, 189.
23. E.R.T. Tiekink, M. Gielen, A. Bouhdid, R. Willem, V.I. Bregadze, L.V. Ermanson and S.A. Glazun, *Metal-Based Drugs*, 1997, **4**, 75.
24. M. Gielen, *Coord. Chem. Rev.*, 1996, **151**, 41.
25. K. Nomiya, N.C. Kasuga, I. Takamori and K. Tsuda, *Polyhedron*, 1998, **17**, 3519.

**Received: January 21, 1999 - Accepted: January 27, 1999 - Received in revised camera-ready format: January 29, 1999**

